# Kind

**Published:** 1909-10

**Authors:** 


KIND.—Dr. W. W. Keen, the great Philadelphia surgeon, kindly points out to the readers of the "Octopus" that "data" is plural, and should not be used with a verb singular. On the train to Mexico City there was a smug, complacent Englishman seated next to a lively young woman from San Antonio. He said to her with a patronizing air, "We have just crossed the Tropic of Cancer." "Have we," said the girl; "I didn't see it or feel any jolt." "It is only an imaginary line," said he, "on the map to mark the boundary of the temperate and the torrid zones." "Thanks, so much," said the girl. "How very interesting. I'll make a note of it." What would Professor Keen say to "septic poisons" and "poisonous toxines" and "uricacidaemia," terms used by a somewhat distinguished writer in a leading journal lately; or to Commander Peary "negotiating" twenty miles?

